# Reliable nanomaterial classification of powders using the volume-specific surface area method

**DOI:** 10.1007/s11051-017-3741-x

**Published:** 2017-02-11

**Authors:** Wendel Wohlleben, Johannes Mielke, Alvise Bianchin, Antoine Ghanem, Harald Freiberger, Hubert Rauscher, Marion Gemeinert, Vasile-Dan Hodoroaba

**Affiliations:** 1 Department of Material Physics, BASF SE, 67056 Ludwigshafen, Germany; 2 BAM–Federal Institute for Materials Research and Testing, 12205 Berlin, Germany; 3 MBN Nanomaterialia s.p.a, 31050 Vascon di Carbonera, Treviso Italy; 4 R&I Centre Brussels, Solvay, 1120 Brussels, Belgium; 5 Nanobiosciences Unit, Joint Research Centre, European Commission, 21027 Ispra, Italy

**Keywords:** Nanomaterial, Classification, Regulation, VSSA, Size measurement, Particle size

## Abstract

The volume-specific surface area (VSSA) of a particulate material is one of two apparently very different metrics recommended by the European Commission for a definition of “nanomaterial” for regulatory purposes: specifically, the VSSA metric may classify nanomaterials and non-nanomaterials differently than the median size in number metrics, depending on the chemical composition, size, polydispersity, shape, porosity, and aggregation of the particles in the powder. Here we evaluate the extent of agreement between classification by electron microscopy (EM) and classification by VSSA on a large set of diverse particulate substances that represent all the anticipated challenges except mixtures of different substances. EM and VSSA are determined in multiple labs to assess also the level of reproducibility. Based on the results obtained on highly characterized benchmark materials from the NanoDefine EU FP7 project, we derive a tiered screening strategy for the purpose of implementing the definition of nanomaterials. We finally apply the screening strategy to further industrial materials, which were classified correctly and left only borderline cases for EM. On platelet-shaped nanomaterials, VSSA is essential to prevent false-negative classification by EM. On porous materials, approaches involving extended adsorption isotherms prevent false positive classification by VSSA. We find no false negatives by VSSA, neither in Tier 1 nor in Tier 2, despite real-world industrial polydispersity and diverse composition, shape, and coatings. The VSSA screening strategy is recommended for inclusion in a technical guidance for the implementation of the definition.

**Electronic supplementary material:**

The online version of this article (doi:10.1007/s11051-017-3741-x) contains supplementary material, which is available to authorized users.

**Graphical abstract:**
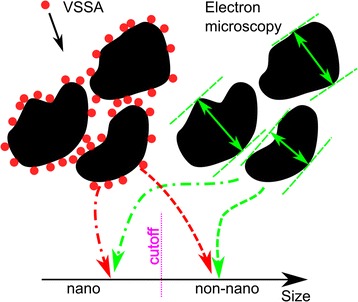
We evaluate the extent of agreement between classification by electron microscopy (EM) and classification by Volume-Specific Surface Area (VSSA) on a large set of diverse particulate substances. These represent the challenges anticipated for identification of nanomaterials by the European Commission recommendation for a definition of nanomaterials for regulatory purposes.

## Introduction

The European Commission (EC) published a recommendation on the definition of nanomaterial (NM) for regulatory purposes (European Commission 2011). According to this recommendation, a material is considered to be a NM if 50% or more of the particles in number metrics have an external dimension between 1 and 100 nm. If this criterion is not fulfilled, the material is classified as non-NM. Furthermore, according to the EC’s recommendation (European Commission 2011) and if requested in specific legislation, materials can be classified as NM if their volume-specific surface area (VSSA) is larger than 60 m^2^/cm^3^. However, in the EC’s recommendation (European Commission 2011), there is no corresponding lower VSSA threshold that would allow materials to be classified as non-NM if their VSSA is below that threshold. Such a criterion would be very helpful, because for the purpose of material registration, classification as NM or non-NM is required (Kreyling et al. 2010; SCENIHR 2010). Consequently, to classify a material as non-NM currently, it must be shown explicitly that less than 50% of the particles have an external dimension between 1 and 100 nm, which in practice can become very difficult (Roebben et al. 2014).

The VSSA is an integral property of materials, and it is obtained by dividing the samples’ external surface (*S*) by its solid volume (*V*) or by multiplying the specific surface area (SSA, surface per mass) by the materials skeletal density (ρ). It is conventionally stated in units of m^2^/cm^3^.

1 $$ \mathrm{VSSA}=\frac{S}{V}=\mathrm{SSA}\times \rho $$

Its value depends on the particle size and size distribution: small particles have a large VSSA and vice versa. The threshold value of 60 m^2^/cm^3^ has a direct relation to the primary (size-based) NM defining criterion as 60 m^2^/cm^3^ is the theoretical VSSA of a material consisting of perfectly monodispersed spherical particles with a diameter of 100 nm (the size-based upper cutoff). Perfectly monodispersed cubic particles with an edge length of 100 nm have the same VSSA of 60 m^2^/cm^3^.

For dry powders (with the appropriate adsorption isotherms of type II or IV (ISO 9277 2010)), the VSSA can be determined via a gas-adsorption measurement of the SSA by the Brunauer–Emmett–Teller (BET) method (ISO 9277 2010; Brunauer et al. 1938; Dabrowski 2001; Hackley and Stefaniak 2013) and multiplying it by ρ from a He pycnometry (DIN 66137-2 2004) measurement. Prior to the He-pycnometry measurement, the sample has to be dried to constant weight in order to avoid irreversible changes of the surface; further, the sample has to be flushed with Helium gas before the measurement is started. Similarly, prior to the gas-adsorption measurement of the SSA, the physically adsorbed materials have to be removed from the sample surface by degassing, again to avoid irreversible surface changes. Gas desorption can be achieved by flushing with an inert gas at elevated temperatures or under vacuum conditions. Recommended processes for degassing are described in detail in ISO 9277 2010. Degassing is usually an implemented step of the BET measurement process in commercial devices. For unknown samples the maximum temperature at which the sample surface structure is not affected has to be determined in a first step, e.g., by thermal analysis or by comparing experiments under different degassing conditions of time and temperature.

What makes this procedure so interesting in comparison to the size-based criterion is that BET is a well-known, low-cost, standardized method, which can be applied on dry powders without further sample preparation except degassing, is agglomeration-tolerant, and leads to reliable results (Hackley and Stefaniak 2013). Furthermore, in many cases it is routinely applied to materials by their manufacturer and therefore, a NM classification based on VSSA data could be performed without the need of additional measurements, provided that VSSA was accepted as valid criterion. Also other experimental techniques are capable of measuring the VSSA, like small-angle X-ray scattering (SAXS) (Radlinski et al. 2004), tomography at an electron microscope (Van Doren et al. 2011), or –under certain conditions– also ultrasound spectroscopy (USSp) (Babick and Richter 2006), but so far they cannot compete with the popularity of the BET method, e.g., due to severe limitations of the accessible size range (SAXS) or due to excessive costs (electron tomography).

On the other hand, determining the particle size distribution for applying the size-based criterion can often be a tedious and more expensive task because adequate sample preparation is a necessary but difficult and time-consuming prerequisite (Babick et al. 2016).

It should be noted that the EC nanomaterial definition builds on the common understanding of VSSA values as defined by Eq. (1), which uses integral material parameters determined from the entire sample, as this is the only VSSA value accessible via the classical gas adsorption methods (e.g., BET). It should not be confused with a *particle-number-weighted* average VSSA also described in the literature (Lecloux 2015). The particle-number-weighted average VSSA cannot be determined experimentally based on ensemble values but would require size-resolved determination of both surface and volume. Such an approach has been pioneered by electron tomography (Van Doren et al. 2011), but then evaluation by the size criterion is a more direct route to classification, and the excessive costs limited the statistics to typically 5, maximally 10 particles per material. A particle-number-weighted VSSA concept does not relate to the specific surfaces determined experimentally by conventional ensemble gas adsorption isotherms with either *t*-plot or BET evaluation (Gibson et al. 2016). In the present paper, we use exclusively the VSSA accessible by gas adsorption measurements (Eq. (1)).

When applying the VSSA method for the positive identification of NMs only (as recommended by the EC (European Commission 2011)) on non-porous materials, the classification can be considered as very reliable. Only few particle shapes exist (e.g., tetrahedral) having a smallest dimension larger than 100 nm and a VSSA > 60 m^2^/cm^3^ (Roebben et al. 2014), for which the VSSA method can lead to a false-positive classification (i.e., a non-NM that is falsely classified as a NM. Analogously, a false negative is a NM which is falsely classified as non-NM). However, even though theoretically possible, these shapes are to the best of our knowledge hardly encountered in industrial materials (Stark et al. 2015). When the particles are porous, the VSSA will be larger than expected from their outer dimensions (due to the additional surface of the pores, which conventional BET cannot distinguish from the external surface) and consequently, such materials should be excluded from the analysis, and the classification should not be done based on VSSA measurements. However, from a consumer perspective, false-positive classifications are uncritical as they trigger a more thorough safety assessment with additional testing (Rauscher et al. 2015). From a producer’s perspective, false positives generate costs beyond the actual requirements and are thus a competitive disadvantage.

The situation is completely different when looking at the classification of non-NMs. Many possibilities exist (Roebben et al. 2014) when materials which are unambiguously NMs according to the size criterion can exhibit a VSSA below 60 m^2^/cm^3^. This can be the case for specific particle shapes, for example, fibers or platelets, having only two or one external dimension between 1 and 100 nm, respectively. Furthermore, polydispersity, multimodality (experimental example in the supporting information (SI)), and aggregation can reduce the VSSA as compared to the median size of the smallest dimension and could consequently lead to false-negative classifications when relying exclusively on a single VSSA cutoff criterion of 60 m^2^/cm^3^. When a high degree of aggregation is present in the material, not the entire primary particles surface is accessible to gas adsorption and hence no reliable result can be expected.

**Fig. 1:**
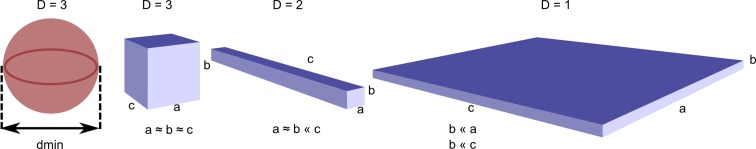
Scheme of prototypical particle shapes, having *D* = three, two, and one small dimensions

As discussed above, when applying the VSSA criterion with a single threshold, several material properties may prevent it from being a reliable tool for the NM classification: particle shape, porosity, aggregation, polydispersity and multimodality. However, for some of these properties, solutions to correct for their effect are available. In order to expand the VSSA concept also to other than spherical particle shapes, the Joint Research Centre (JRC) has introduced a shape-dependent cutoff value (Roebben et al. 2014):

2 $$ \mathrm{VSSA}\ \mathrm{cutoff}=60\frac{{\mathrm{m}}^2}{\mathrm{c}{\mathrm{m}}^3}\times \frac{D}{3} $$

with *D* the number of small particle dimensions (i.e., *D* = 3 for spherical and roughly spherical particles, *D* = 2 for needles, fibers and tubes, and *D* = 1 for platelets, see Fig. 1), which allows for NM classification also for non-spherical particles—but only with pre-knowledge on their shape. Further, the pore surface can be separated from the outer particle surface by a detailed analysis of the full adsorption isotherm, e.g., using an appropriate *t*-plot method (Lippens and de Boer 1965; ISO 15901-3 2007; Lecloux 1981, 1986; Schneider 1995; Galarneau et al. 2014).

In this work, a quantitative relation between the VSSA and the particle size as measured by electron microscopy (EM, as a general term for scanning electron microscopy, SEM and transmission electron microscopy, TEM), using the available VSSA corrections for particle shape and porosity, will be demonstrated for the first time on a set of representative industrial materials. These materials are part of the European research project NanoDefine (http://www.nanodefine.eu) and a joint study by the JRC and Eurocolour (Pena et al. 2014). In the case of NanoDefine, the materials were selected to cover most kinds of sizes, shapes, manufacturing processes, and chemical compositions relevant in industry, whereas the materials from Pena et al. (2014) are representative for the pigments and fillers on the market. Consequently, the results can be expected to be representative for a broad range of industrial materials.

Furthermore, a VSSA-based NM screening strategy, capable of identifying NM and non-NM for the purpose of the EC recommendation, is proposed and tested on an additional set of industrial materials.

## Materials and data

**Fig. 2:**
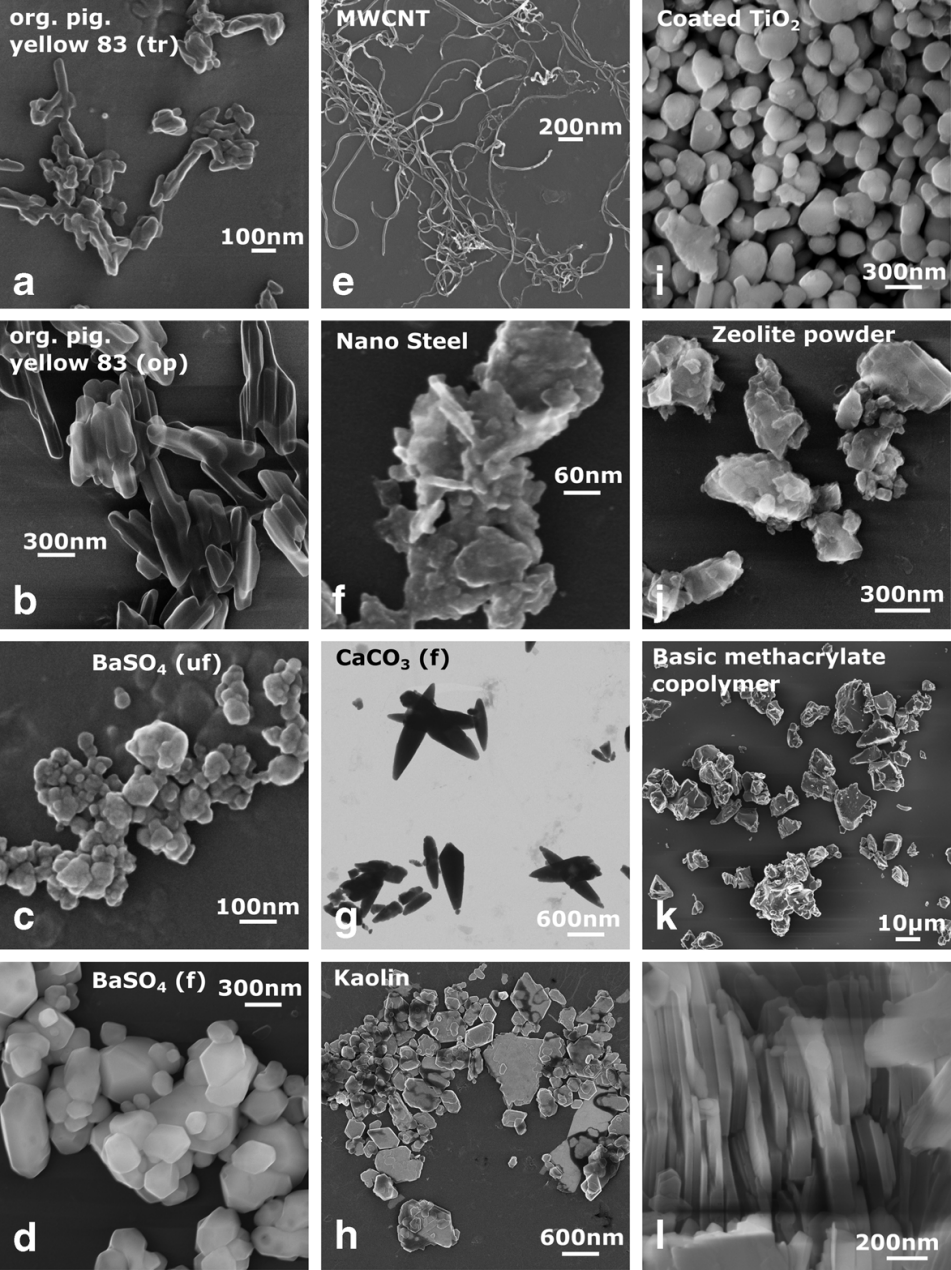
SEM images of the NanoDefine materials. **a**, **b** Organic pigment yellow 83 as transparent and opaque pigments, respectively, **c**, **d** BaSO_4_ in ultrafine and fine grade, respectively, **e** multi-walled carbon nanotubes (MWCNT), **f** nanosteel, **g** CaCO_3_, fine grade, imaged in transmission-SEM (TSEM) mode, **h** kaolin, **i** coated TiO_2_, **j** zeolite powder, ZSM-5, **k** basic methacrylate copolymer (carbon coated for imaging), **l** upright standing kaolin. Further details can be found in the supporting information

While the materials used for the JRC–Eurocolour study are already described in the public literature (Pena et al. 2014), the materials from the NanoDefine project are presented in Fig. 2 and will be discussed in the following. Information about the materials’ VSSA values, for both groups of materials, can be found in the SI, including an evaluation of the measurement precision.

SEM images of the NanoDefine materials are shown in Fig. 2. All of the materials contained therein are of high industrial relevance and are produced in considerable amounts. The materials were selected to possess various properties in order to challenge the available particle sizing techniques employed for NM classification (Babick et al. 2016). There are particles of different sizes, such as the organic pigment yellow 83 which is produced in a transparent version (a) and as an opaque pigment (b). Another pair of materials with the same chemical composition but different sizes are the two forms of BaSO_4_, having an ultrafine (c) and a fine (d) grade. Different particle shapes are also represented: There are compact particles (roughly spherical) such as the two BaSO_4_ materials (c, d) and the coated TiO_2_ (i), particles with an elongated shape like the organic pigment (a, b), the carbon nanotubes (e), and the CaCO_3_ particles (g) as well as platelet particles like the nanosteel (f) and the kaolin (h). Various examples exist for organic (a, b, e, k), inorganic (c, d, g, h, i, j), and metal particles (f). Furthermore, there is one example for a core-shell particle (i) and two examples for porous particles (i, j).^1^ All of the materials have a polydispersity of 20%–60% (standard deviation of particle size distribution (PSD) in relation to median size), which is typical for industrial, shape-engineered particles (see Table 1). Note that for the pigments and fillers in the present study, the shape and size are essential parts of the product specifications, as they have direct relevance for the intended functionality. For other particulate intermediates, whose function does not depend on size and shape, e.g., because they are melted or dissolved in the final product, shape and polydispersity tend to be far less controlled.

**Table 1:** VSSA (by BET) and EM results of NanoDefine materials. The particle size distribution (PSD) determined by SEM was used to derive rough measures for the materials polydispersity. It is expressed by the ratio of the PSDs standard deviation to the median particle diameter and is stated in percent

Material	*SSA* (BET) (*n* = 3)	SSA (BET) StD	Skeletal density	VSSA	Median Feret_min_ (EM)	Median Feret_min_ (EM) StD	Polydispersity (EM)
	m^2^/g	m^2^/g	g/cm^3^	m^2^/cm^3^	nm	nm	(%)
Organic pigment (transparent)	67.6	4.7	1.5	100.4	39.8	0.5	30
Organic pigment (opaque)	17.4	0.8	1.5	26.1	188.8	31.8	55
BaSO_4_ (fine grade)	2.5	0.5	4.4	11.1	248.9	34.9	56
BaSO_4_ (ultrafine grade)	36.9	0.4	4.4	162.3	27.3	6.6	52
MWCNT	252.5	17.0	2.1	517.6	12.1	0.5	18
Nanosteel	9.6	1.0	5.1	49.3	155.0	93.0	76
CaCO_3_ (fine grade)	5.8	0.1	2.7	15.4	156.9	3.3	52
Kaolin	16.0	0.4	2.6	41.9	123.8	4.2	135
Coated TiO_2_	14.8	0.3	4.0	58.9	183.7	2.1	32
Zeolite powder	388.0	50.8	2.1	803.2	118.2	14.9	84
Basic methacrylate copolymer	1.3	0.1	1.1	1.5	2014.0	0.0	70

For all NanoDefine materials, standard BET was measured by three independent labs, and the skeletal densities were determined by He pycnometry in compliance to DIN 66137-2 (2004). The BET standard procedure was defined as five-point BET (relative pressure: 0.05; 0.1; 0.15; 0.2; 0.3); two measurements were performed for each sample. Preparation of inorganic materials was performed by degassing at 150 °C for 3 h in air and further storage in a desiccator before measurements of density or of specific surface. These thermal conditions were sufficient to achieve constant weight. Sensitive organic samples were processed by degassing at 50 °C for 8 h in air and then stored in a desiccator before measurement. Prior to BET measurement, an additional degassing of the inorganic samples inside of the BET devices for 3 h at 150 °C in nitrogen atmosphere was performed; sensitive organic samples were treated at 50 °C for 3 h in nitrogen atmosphere, respectively.

Only in the case of the BaSO_4_ materials, the literature value of 4.4 g/cm^3^ was used because it was considered to be more plausible. The measured density value of BaSO_4_ (fine grade) showed an average of 4.28 g/cm^3^ and of BaSO_4_ (ultrafine grade) 4.01 g/cm^3^, respectively. Deviations of this order of magnitude between the literature value and measured values may be caused by insufficient thermal pretreatment period of the samples before density measurements. These slight differences of below 10% do not cause any significant effect on the associated VSSA values and on the derived dmin_VSSA_ values, so that the classification of the two BaSO_4_ materials remains unaffected. The median minimum Feret diameter was extracted for all NanoDefine materials (except for the nanotubes, where the tube diameter was measured) from SEM micrographs and additionally, for some of the materials, one or two independent TEM results are available.

For the materials from the JRC/Eurocolour study, BET was measured by eight independent labs, the skeletal density and a single TEM evaluation of the particle size were provided by the manufacturer. Due to insufficient data quality in the EM evaluation, the Al-Co-Blue material was excluded from the presented study. All available data was averaged and can be found in Table 2; more details are provided in the supporting information, specifically: Derivation of the quantity dminVSSA; Calculation of the uncertainty introduced by the aspect ratio cutoff values; Calculation of multimodal material VSSA; EM data for NanoDefine materials, including SEM method on platelets; EM, BET, density data for the JRC/Eurocolour materials; EM, BET, density data for the further real-world industrial materials.

**Table 2:**
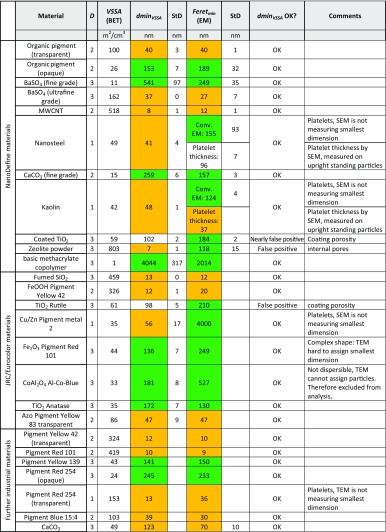
Quantitative relation between dmin_VSSA_ and the minimum Feret diameter from electron microscopy images (for the MWCNTs, the tube width was measured). *D* refers to the number of small dimensions and the dmin_VSSA_ was calculated according to Eq. (3). The color code highlights agreements and discrepancies with respect to the classification as NM (*yellow*) or non-NM (*green*). The standard deviations on BET and thus on VSSA stem from independent measurements of the ensemble average from different labs: *n* = 8 different BET labs for Eurocolor/JRC; *n* = 3 different BET labs for NanoDefine. The standard deviation on EM stems from independent measurements on *n* = 1–3 labs for NanoDefine. The Eurocolor/JRC materials and further industrial materials were measured by TEM only by a single lab, with full results shown in the SI

## Results

For each of the NanoDefine materials (Fig. 1), three independent BET values are available. Two of them were measured in the laboratories of project partners (in compliance to ISO 9277 2010, the third value was provided by the material manufacturer. The resulting average values and standard deviations (StD) are reported in Table 2, including the conversion to VSSA by Eq. 1.

For kaolin, an outlying BET value of the manufacturer was removed from the analysis and replaced by a replicate measurement carried out by one of the project partners. These agree within 2.5% relative standard deviation. BaSO_4_ (fine grade) has a relative standard deviation approaching 20%. Although this may be linked to its low SSA, another low-surface-material (CaCO_3_) has a relative standard deviation below 3%. Project internal replicate measurements identified different evacuation times as source of the 15% standard deviation for the microporous zeolite powder.

The skeletal densities were determined by He pycnometry in compliance to DIN 66137-2 2004. The only exceptions are both BaSO_4_ materials, for which the literature value of 4.4 g/cm^3^ was used, because it was considered to be more plausible.

The median minimum Feret diameter was extracted for all materials from SEM micrographs and additionally, for some of the materials, one or two independent TEM results are available. All available EM data was averaged and can be found together with the resulting standard deviation in Table 1. The sample preparation and imaging parameters for all EM measurements are given in Table S2.

We now use the shape-specific concept of Eq. (2) and Fig. 1 to establish a quantitative relationship between the VSSA and the smallest particle dimension in the following way (with dmin_VSSA_ in micrometer when entering the VSSA in m^2^/cm^3^):

3 $$ {\mathrm{dmin}}_{\mathrm{VSSA}}(D)=\frac{2 D}{\mathrm{VSSA}} $$

When entering the VSSA in m^2^/cm^3^, dmin_VSSA_ is obtained in micrometer. A detailed derivation of this quantity is presented in the SI. If in doubt, the prevailing particle shape, and hence the value for *D*, can be determined by a descriptive SEM scan without the need to fully de-agglomerate the particles, thus saving time, both on the sample preparation and on the statistical analysis.

In the present paper, the dmin_VSSA_ is compared with the median of the minimum Feret diameter or the tube width for multi-walled carbon nanotubes (MWCNTs) (size-based NM criterion), obtained from evaluating a large number of particles in electron microscopy (EM) images (Table 2). For practical reasons and because it is not explicitly specified by the JRC, particles with an aspect ratio < 3:1 will be considered as spherical (*D* = 3), particles with an aspect ratio > 3:1:1 are classified as fibers (*D* = 2), and particles with an aspect ratio > 3:3:1 as platelets (*D* = 1). An evaluation of the influence of these aspect ratio cutoffs on the dmin_VSSA_ can be found in the SI, and results for hypothetical shapes that are limiting cases of the shape categories in a deviation from −44% to +67%. This fact will be accommodated in a screening strategy to be developed at the end of this paper.

In absence of pre-knowledge on the particle shape, one can assume *D* = 3 as default (roughly spherical particles). In this situation, only an equivalent sphere diameter (*d*_VSSA_) can be calculated from the VSSA, which does not necessarily correspond to the smallest particle dimension:

4 $$ {d}_{\mathrm{VSSA}}=\frac{6}{\mathrm{VSSA}} $$

With such an assumption, the maximum deviation to the smallest particle dimension dmin_VSSA_ occurs for the case of *D* = 1, resulting in a *d*_VSSA_ which is too large by a factor of 3.

Table 2 contains the EM and VSSA data from the NanoDefine and JRC/Eurocolour materials, as well as their shape parameter *D* and the derived dmin_VSSA_. One part of the parameters was measured by three independent laboratories, another part by eight independent laboratories. The results were averaged over the different laboratories, and the standard deviation obtained this way is also given in Table 2. As a quick visual representation of the classification result, materials classified as NM (size ≤100 nm by either method) are marked with yellow, non-NM with green.

**Fig. 3:**
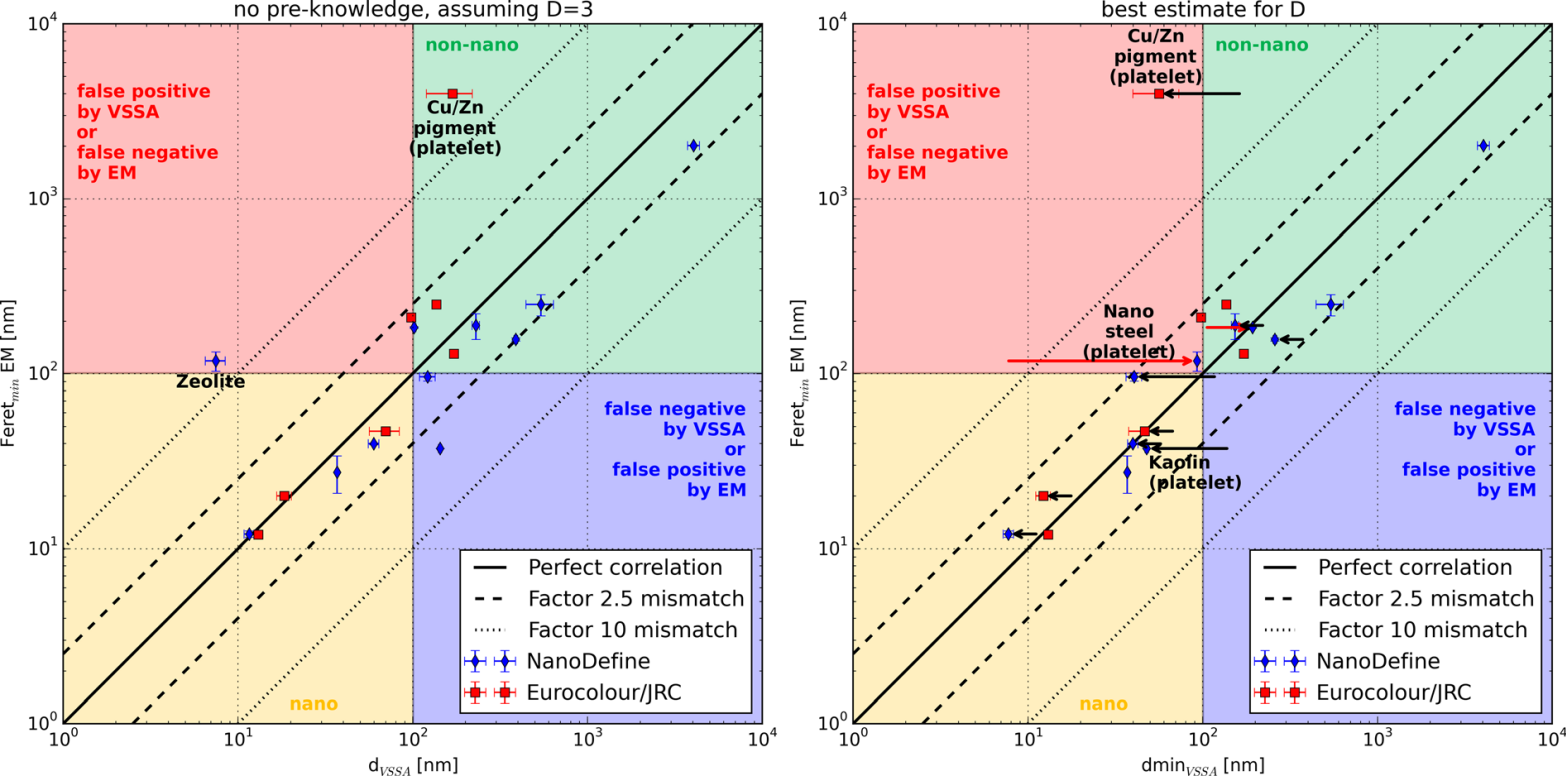
Correspondence between the VSSA-derived particle dimension *d*_VSSA_ and dmin_VSSA_ and the Feret_min_ from EM. In the *left panel*, the available data from the NanoDefine and the JRC/Eurocolour materials are shown, assuming the case of no pre-knowledge on the particle shape (i.e., *D* = 3 for all materials and no porosity correction). In the *right panel*, the same materials are shown, but this time dmin_VSSA_ was calculated using the best estimate for the number of small dimensions *D* (the shift which occurred in comparison to the case with *D* = 3 is indicated with *black arrows*), and the effect of an additional *t*-plot evaluation (always ISO method except for TiO_2_ which is calculated according to Lecloux) is shown by *red arrows*. In both panels, a color code indicates the NM classification of the data points. In the *yellow and green areas*, the materials are consistently classified as nano and non-nano, repectively, by VSSA and EM. In the *red and blue areas*, both methods disagree on the classification. The *error bars* are the standard deviations resulting from size measurements (*d*_50_ for EM and dmin_VSSA_, respectively) of different labs. Note that these are no combined measurement uncertainty budgets, which is particularly important in the case of platelet materials; as for them, EM cannot assess the smallest particle dimension

In most cases, the materials are classified consistently by EM and VSSA. This is a major result that was not initially expected for this diverse set of substances, shapes, sizes, and products in processes. The remaining discrepancies can be attributed to specific material properties that induce misleading results for one of the measurement techniques. In several cases, conventional EM cannot address the smallest dimension of platelet materials as it measures two-dimensional projections of flat particles, lying parallel to the substrate. For the NanoDefine platelet materials (kaolin and nanosteel), a value for the platelet thickness could be generated by SEM imaging of randomly oriented platelets (Fig. S2, methodical SEM details in the SI). Both EM sizes are given in Table 2 and it is evident that the measured platelet thickness agrees much better with the dmin_VSSA_ than the result from a conventional EM evaluation. For the kaolin a good agreement between the platelet width measured by SEM of 37.4 nm and the dmin_VSSA_ (*D* = 1) of 48 nm is observed. For the nanosteel, there is a considerable uncertainty not only in the conventional EM evaluation but also in the evaluation of the platelet thickness due to the difficulty to identify primary particles and distinguish them from the particles surface structure. Furthermore, the particle shape is rather inhomogeneous and thus it is very challenging to provide a reasonable EM measurement. For such kinds of materials, a classification using the size criterion can become very difficult, and the result will be to some extent arbitrary. Classification by VSSA only, on the other hand, could be the only reliable option for such difficult materials. In Figs. 3 and 5, the SEM platelet thickness of these two materials is used as EM size exclusively.

All cases of discrepancies can be understood by the mentioned material properties as follows: All platelet materials (nanosteel, kaolin, Cu/Zn pigment metal 2) are classified as NM by VSSA, which is supposed to be correct, but the *d*_50_ (median diameter) measured with conventional EM is much larger than 100 nm and would classify the material as non-NM. As discussed above, this effect is observed because conventional EM in those cases does not measure the particles’ smallest dimension. All remaining cases of discrepancies with respect to the classification (coated TiO_2_, zeolite powder, coated TiO_2_ rutile) are due to particle porosity. Classification by VSSA is challenged by porous particles because for them, the measured surface area does not only consist of the outer particle surface but also of the pore surface.

The *t*-plot method claims to be capable of separating the surface contributions of the outer particle surface and of the internal pore surface (Lippens and de Boer 1965; ISO 15901-3 2007). To apply this method, the adsorption isotherm needs to be recorded from pressures << *p*_0_/10, which requires more effort than necessary for standard BET. Several versions of the *t*-plot method exist (Lippens and de Boer 1965; ISO 15901-3 2007; Lecloux 1981; Lecloux et al. 1986; Schneider 1995; Galarneau et al. 2014). In this work, two different forms of the *t*-plot method are applied to all NanoDefine materials and their performance is compared: *t*-plot as standardized in ISO 15901-3 (ISO 15901-3 2007) and directly integrated in most BET instrument software and, within the framework of a collaboration between the projects NanoDefine and NANoREG, *t*-plot as described by Lecloux (Lecloux 1981; Lecloux et al. 1986), promising a better performance as compared to the standardized method by optimized fitting of the low-pressure range of the isotherms.(Lecloux A, personal communication; 2015).

In Table 3, the different values for the total surface, the surface of the micropores, and the external surface are compared for both *t*-plot methods. Furthermore, for both external surface values, the corresponding dmin_VSSA_ is calculated and compared to the result from the conventional BET evaluation and EM. For most materials, no significant discrepancies are observed between the *t*-plot and the BET evaluation, which does not come unexpectedly for non-porous particles. For the materials for which BET and *t*-plot lead to deviating results, the numbers are printed in *italic* face and, by comparison with the EM data, reliable *t*-plot results are colored in green and questionable ones in red.

**Table 3:**
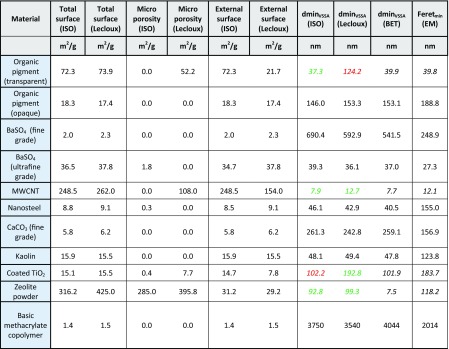
Separation of the external particle surface and the pore surface by isotherm evaluation with the *t*-plot method according to ISO 15901-3 (ISO 15901-3 2007) and to Lecloux (Lecloux 1981, 1986) on the NanoDefine materials (Lecloux A, personal communication; 2015). The total surface, the surface of the micropores and the external surface are compared for both methods and finally, the dmin_VSSA_ derived from both values for the external surface area are compared to the one obtained by conventional BET and the minimum Feret diameter from EM measurements (and the tube width for MWCNT). In case of a significant difference between BET and *t*-plot, the numbers are printed in *italic* and reliable (by comparison with EM) results are colored in *green*, questionable results in *red*

In the case of the transparent organic pigment, which is not expected to be porous, the value from the ISO *t*-plot (37.3 nm) is in good agreement with the BET result (39.9 nm) and the EM evaluation (39.8 nm). However, the method by Lecloux leads to a much larger dmin_VSSA_ (124.2 nm). The Lecloux method would thus generate a false negative classification, which cannot be accepted as part of a screening strategy. In the case of the MWCNTs, the *t*-plot evaluation by Lecloux (12.7 nm) yields a better fit with the EM *d*_50_ (12.5 nm) than the *t*-plot evaluation according to ISO (7.9 nm). Apparently, gas adsorption occurs also at the inner surface of the MWCNTs, which is detected as pore surface by Lecloux’s *t*-plot method. Nevertheless, the classification of MWCNT (as a NM) by both methods is unambiguous. For the coated TiO_2_, where pores are known to be present in the alumina-based coating, the ISO method fails to detect these pores, but they clearly appear in the method by Lecloux. According to the latter, a dmin_VSSA_ of 192.8 nm can be derived, which is in close agreement with the EM *d*_50_ of 183.7 nm. For the zeolite powder, both *t*-plot methods detect the internal pores and give similar results which agree very well with the value from EM. Of note, both *t*-plot results originate from the identical raw data isotherm and are merely different fits in the low-pressure range, where the ISO method is standardized, whereas the Lecloux method is optimized per material. The differences might reflect different micropore shapes, micropore connectivities, physisorption effects, etc. but cannot be clearly attributed here.

As a result of this evaluation, the *t*-plot method is capable of NM classification of porous materials by VSSA. For all porous NanoDefine materials, an adequate solution could be found, where the particles’ external and pore surface could be separated correctly, leading to a dmin_VSSA_ which is in good agreement with the EM *d*_50_. However, care needs to be taken when applying the *t*-plot method because both presented methods have deficiencies with some of the materials. The ISO method is more reliable for the organic pigment, whereas the method by Lecloux is more appropriate in the case of MWCNTs and the coated TiO_2_. For zeolite, both methods are successfully applicable. Which method to select, consequently, depends on the particular material under investigation, but only the case of coated TiO_2_ really benefits from the Lecloux *t*-plot method for improved correctness of classification.

A summary for the representative NanoDefine and JRC/Eurocolour materials can be found in Fig. 3, where the minimum Feret diameter from EM (tube width for MWCNT and platelet thickness for kaolin and nanosteel) is plotted against their *d*_VSSA_ (left panel) and dmin_VSSA_ (right panel) in order to compare their agreement. Both panels use the same color code to describe the resulting classification: yellow for NM, green for non-NM, red and blue for an inconsistent classification between the two techniques. Furthermore, solid black lines indicate a perfect quantitative correlation between the results of the two techniques, the broken lines mark a mismatch of factor 2.5, and the dotted lines represent a mismatch of factor 10. The left panel illustrates the correspondence between EM and *d*_VSSA_ for the naïve case when all particles are assumed to be spherical (*D* = 3). In the right panel, the number of small dimensions was considered adequately and the resulting shift as compared to the evaluation with *D* = 3 is indicated by horizontal black arrows. Furthermore, the effect of a potential *t*-plot evaluation on the NanoDefine materials (using the ISO t-plot result from Table 3 except for coated TiO_2_, using the Lecloux t-plot result) is indicated by red arrows.

It can be observed that when both corrections are applied (right panel), both methods agree on most data points within a factor of 2.5. The only point which lies outside this range is the platelet material Cu/Zn pigment, where a conventional EM assessment measured lateral dimension of the platelets and not the relevant thickness. Remarkably, even the zeolite displays good agreement to the EM value after application of the *t*-plot method.

This good overall agreement to within a factor of 2.5 between EM and dmin_VSSA_ is observed despite the presence of polydispersity (20%–60%), organic, inorganic, metal-organic, carbonaceous, metallic substances, and irregular shapes. It should be noted that several of the materials of the present study are considerably aggregated: fumed silica, BaSO_4_, and the organic pigment, and most of the other ultrafine (nano) particles show a significant aggregation, in agreement with the EM micrographs. It seems that for industrially relevant materials, as studied here, the effects of aggregation and polydispersity are within the uncertainty of the correlation between the two very different measurement principles. Severe aggregation (sintering) and bimodality are extreme cases to be discussed below.

Classification strategy

Based on the good agreement between the dmin_VSSA_ and reliable EM data within a factor of 2.5, observed for the NanoDefine and the JRC/Eurocolour materials as training set, the authors propose the following VSSA-based NM screening strategy, which can identify NM and non-NM. Note that materials with a VSSA > 60 m^2^/cm^3^ and thus a *d*_VSSA_ < 100 nm can already be directly identified as NM according to the current EC recommendation (European Commission 2011) and are not part of the screening strategy.

1. Measure skeletal density and standard BET
  - Remark: If porosity is expected to be present in the material, try to separate external and pore surface by appropriate procedures (the ISO *t*-plot method as default, but the Lecloux *t*-plot method for coated TiO_2_). If not successful, escalate to confirmation methods (EM or other).
2. If *d*_VSSA_ > 1000 nm (unknown number of small dimensions: assuming spherical): classify as non-NM.
  - Reason: The maximum deviation between *d*_VSSA_ and dmin_VSSA_ due to the unknown particle shape is a factor of 3. Furthermore, a mismatch between dmin_VSSA_ and EM by not more than a factor of 2.5 was observed in the training set. Combining these two effects, the overall disagreement should be maximum a factor of 7.5. Thus, using 10 times the size-based cutoff (10 × 100 nm = 1000 nm) may be considered as conservative.
3. If *d*_VSSA_ is in the range between 100 and 1000 nm: Take a descriptive SEM image to determine *D* and check for the possible presence of severe aggregation and multimodality.
  - If dmin_VSSA_ < 100 nm using the best estimate for *D*, classify as NM.
  - If dmin_VSSA_ > 250 nm using the best estimate for *D*, classify as non-NM.
    i. Reason: A mismatch between dmin_VSSA_ and EM within a factor of 2.5 was observed in the training set. Hence 250 nm can be considered as conservative.
  - If 100 nm ≤ dmin_VSSA_ ≤ 250 nm using the best estimate for *D*: Borderline region, classify material with confirmation method (EM or other).
  - If multimodality is detected, classify material with confirmation method (EM or other).

It should be kept in mind that in the EC recommendation (European Commission 2011), the size-based criterion has a higher priority than the VSSA. Consequently, if there is doubt about the correct classification, it is always possible to use a confirmation method which directly measures the smallest particle dimension to override the results obtained by the here proposed VSSA screening procedure.

**Fig. 4:**
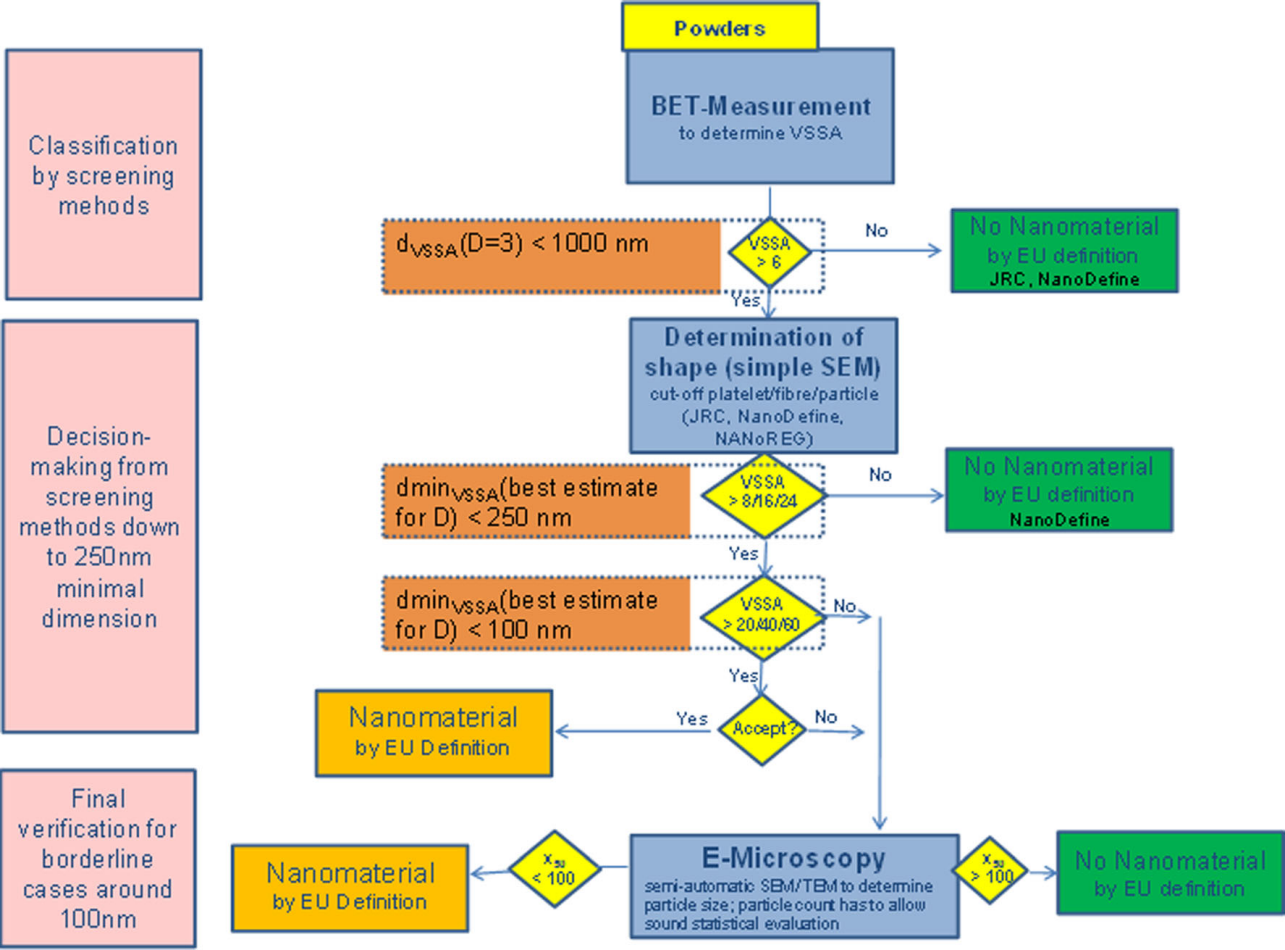
Flow chart of the proposed screening strategy, as proposed in the NanoDefine project (http://www.nanodefine.eu, deliverable D7.10) with the cutoff values adapted to the here developed screening strategy. The *orange boxes* were added to show the correspondence of the dmin_VSSA_ and the shape-dependent cutoff values

**Fig. 5:**
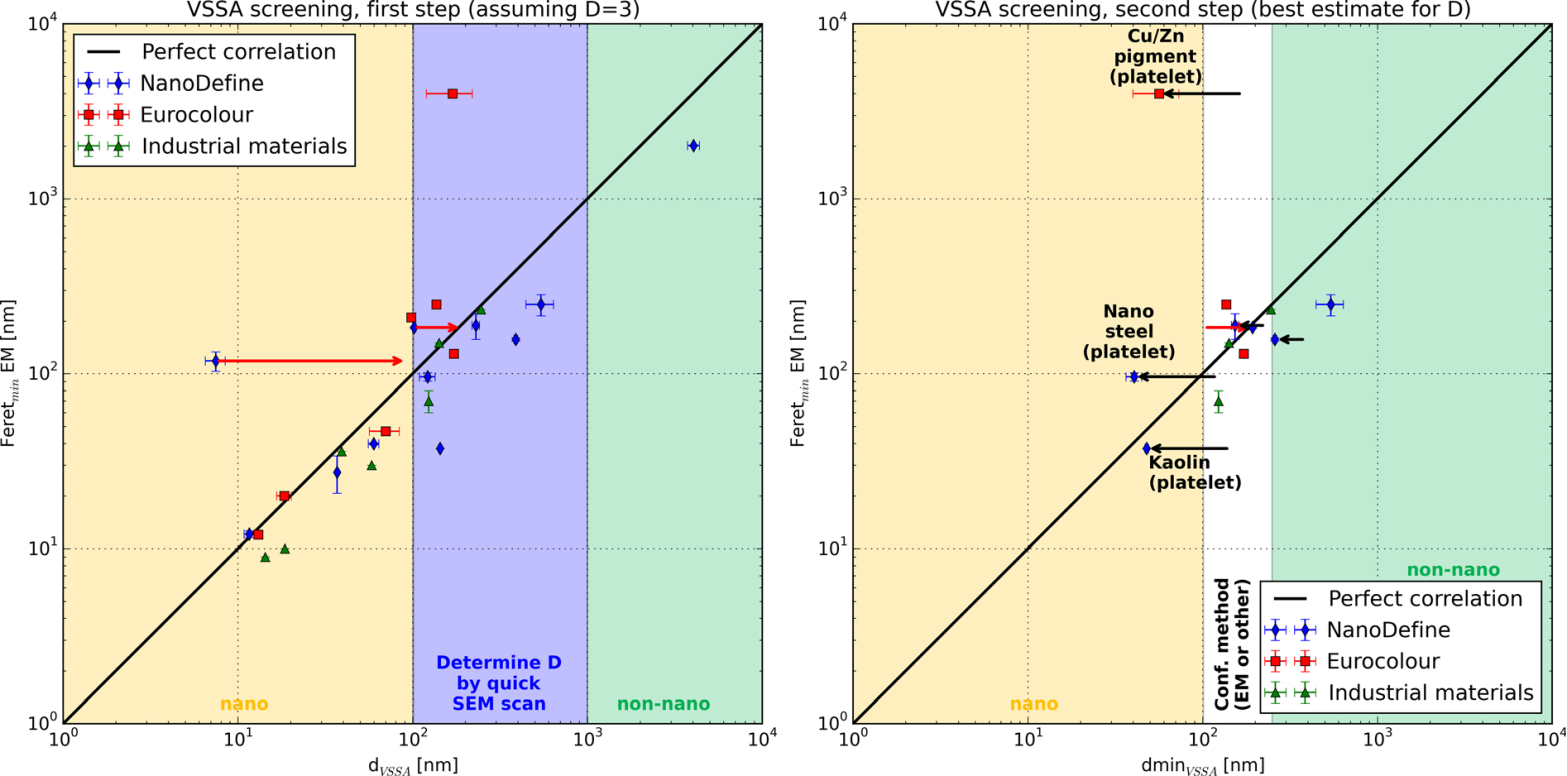
Test of the VSSA screening strategy. Plots similar to the ones in Fig. 2, but with colored background for the VSSA-derived NM classification, and adding the test set of further industrial materials. The *left panel* shows the first step where materials in the *yellow* (nano) and *green* (non-nano) areas are classified without detailed knowledge about the particle shape. Only for the materials in the *blue area*, a descriptive SEM evaluation determines the number of small dimensions. In the *right panel*, the remaining materials (from the *blue area* on the *left*) are classified with the best estimate for *D*, only the ones in the *white area* remain for a detailed evaluation with a confirmation method. As in Fig. 3, *red arrows* indicate the correction by the *t*-plot method and *black arrows* the correction after evaluating the materials with the best estimate for the number of small dimensions

The procedure as presented above is visualized in Fig. 4 as a flow chart. It does not only correctly classify all materials from the training set (NanoDefine and JRC/Eurocolour), but its performance was additionally assessed on another test set of industrial filler and pigment materials. Their VSSA and EM are summarized in the bottom of Table 2, with full BET and EM data documented in the SI (Figure S2, Table S5). In Fig. 5, the screening strategy is applied to all materials available in this study. In the first step (left panel), the materials are evaluated without taking their shape into account (*d*_VSSA_, *D* = 3) and classified according to the procedure as NM or non-NM if possible (yellow and green area, respectively). For the remaining materials (in the blue range), a descriptive SEM scan identifies their shape and is taken into account in the right panel by applying the best estimate for *D* and calculating dmin_VSSA_. Now, further materials can be classified. Only the ones remaining in the white region will require a more detailed analysis by a confirmation method. Of note, the BET and density values of the additional industrial filler and pigment materials were extracted from the manufacturers’ material data sheets without any data generation and thus implement a low-cost screening.

Out of the 25 materials, the proposed VSSA-based procedure classifies 15 as NM, 3 as non-NM, and leaves 7 borderline materials for a more detailed analysis with a confirmation method. Notably, and strongly supporting the proposed screening strategy, no false-negative and only two false-positive classifications were obtained, even within the borderline region. The false positives were the NanoDefine zeolite and the JRC/Eurocolour TiO_2_ rutile material. For both materials, the false-positive classification is due to their porosity. In the case of this TiO_2_, the material is known to have a porous alumina-based surface coating; however, as its BET value is taken from the literature (Pena et al. 2014) and the material was not available to the authors, the *t*-plot method could not be applied to correct for the surface porosity. Interestingly, this material has very similar properties to the TiO_2_ from NanoDefine (see Table 2) and might therefore be the same material. For the NanoDefine TiO_2_, the *t*-plot method could resolve the observed discrepancy. For the case of the zeolite powder, when applying the *t*-plot method (by Lecloux) a dmin_VSSA_ of 99.3 nm is obtained, which is just below the cutoff. The EM evaluation yields a median Feret_min_ of 118 nm with a standard deviation of 15 nm. Consequently, this material is a borderline case, where the classification as NM or non-NM is ambiguous also by EM. Hence, the classification as a NM is the conservative choice in this case.

There are discussions on the consistency between the VSSA and the size criterion of the EC definition recommendation, especially with increasing polydispersity. Increasing the polydispersity of a PSD while keeping its median size constant will, in general, increase the particle-number-weighted VSSA (Lecloux 2015) but decrease VSSA by Eq. (1) as measured by gas adsorption (Roebben et al. 2014). Experimentally, correlation between VSSA and TEM has been confirmed also in a recent report on three TiO_2_, one carbon black, and three SiO_2_ from the OECD sponsorship program, all of compact shape with *D* = 3 in our terminology (Temmerman et al. 2014). Based on a comparison in VSSA metrics, the agreement in this study was well below the factor 2 range. Unfortunately, the pioneering papers by Mast et al. on the derivation of VSSA from TEM and from electron tomography datasets do not specify how the number metrics tomography was converted to ensemble VSSA (Van Doren et al. 2011; Temmerman et al. 2014). As discussed by Lecloux (Lecloux 2015), summing up the surface-to-volume ratio of all particles results in particle-number-weighted VSSA which is inherently still in number metrics (Gibson et al. 2016).

As a first step to simplify the consistency discussion, we note that the priority of size in the EC nanomaterial definition suggests that size determined by EM and the size derived from VSSA should be compared; one should not compare VSSA derived from EM with measured VSSA. If we calculate dmin_VSSA_ based on the BET values published by Temmerman et al. (Temmerman et al. 2014), the maximum deviation to median *D*_p_ (EM) is 40% and is on average +2% across their seven materials. In contrast, their so-called “measured VSSA,” which we would designate as “particle-number-weighted VSSA derived from TEM,” deviated a maximum of 60% from the VSSA determined by BET and was biased with average +13% across their seven materials. Thus, the comparison of sizes, not of surfaces, simplifies measurements and enhances consistency.

As a second step and in order to ground the debate on the metrics that apply for VSSA determined by adsorption isotherms and to illustrate the consequences of real bimodality for VSSA screening, we measured a bimodal mixture. A sample of BaSO_4_ fine was spiked with 10% g/g of BaSO_4_ ultrafine. The particles of both materials are approximately spherical (*D* = 3, see Table 2 and Fig. 2), the ultrafine grade is a clear NM according to the size criterion with a median Feret_min_ of 27 nm, and the fine grade is clearly a non-NM with a median Feret_min_ of 249 nm. Because of the number ratio of 300:1, the mixture has to be a NM according to the size criterion. Three independent BET measurements were performed on the mixture by two different labs and resulted in a mean VSSA of 23 m^2^/cm^3^ with a standard deviation of 3.3 m^2^/cm^3^, which leads to a dmin_VSSA_ of 258 nm, and therefore would falsely classify the material as non-NM.

This effect was predicted earlier by calculated examples and is now experimentally confirmed (Roebben et al. 2014) and demonstrates that multimodal *mixtures* of nano- and non-nano-materials very likely lead to false negative classifications. Hence, such materials need to be excluded from the VSSA screening strategy, whereas typical industrial materials with 70% *polydispersity* (Table 1) were correctly classified (Table 2). More importantly, the measured VSSA values for the mixture of 23 m^2^/cm^3^ (three replicates, two labs) are in excellent accord with the value of 24.6 m^2^/cm^3^ predicted from the TEM size distributions of the individual materials according to Eq. 1 and as described by JRC (Roebben et al. 2014) but are significantly different from the prediction of 309 m^2^/cm^3^ obtained from the same TEM size distribution by a particle number-weighted approach (Lecloux 2015). The calculations are documented in detail in the electronic supplementary information. The same is true for a 50% g/g mixture. We conclude that the mass-based VSSA approach (Roebben et al. 2014) is equal to the VSSA that is measurable by adsorption isotherms, such as from standardized BET.

It might be argued that, with the proposed VSSA screening strategy, heavily aggregated materials will lead to false-negative classifications if in the first screening step the *d*_VSSA_ is larger than 1000 nm. This is true; however, due to the uncertainty margin of a factor of 10 with respect to the size-based cutoff (10 × 100 nm = 1000 nm), the primary particles of such a material would have lost at least 90% of their surface due to the aggregation. One might consider that the particulate nature is lost by such a near-complete sintering, and the material has transformed into a bulk solid with internal nanostructures (Roebben et al. 2014) (Rauscher et al. 2015).

## Conclusions

In this work, the potential of the VSSA method as a classification tool, both for the identification of NM and of non-NM, was evaluated on real-world industrial materials by comparison with results from EM evaluation. When deriving the average size of the smallest particle dimension from VSSA, taking into account the approximate particle shape (sphere, fiber, platelet), a good agreement to within a factor of 2.5 was obtained with the EM results. Achieving such a good agreement is also possible for porous particles using the *t*-plot method, which is capable of separating the pore surface from the particles external surface. Platelet materials are a special case, where the VSSA approach yields a more reliable classification than conventional EM, because EM cannot always address the relevant smallest dimension of the particles.

A VSSA-based NM screening strategy was developed and tested on further industrial materials. During the testing, no false-negative and two false-positive classifications were observed. One of them occurred because the strategy could not be applied entirely (material not available for applying the *t*-plot method) and the other one is a borderline case also for EM.

It is expected that both the observed quantitative agreement between the VSSA-derived size and the median Feret_min_ from EM, as well as the screening strategy, will be helpful for the reliable, fast, and cost-efficient identification of NM and non-NM according to the EC recommendation (European Commission 2011).

## Electronic supplementary material

ESM 1 (DOCX 2598 kb)

## Footnotes

1 Particle coatings obtained from solutions tend to be porous, which is difficult to avoid regardless whether the coating layer is metal oxide, polymeric, or metallic (Iler 1979; Ayral et al. 1998). The porosity of the coatings on titanium dioxide is mainly dealt with in the patent literature (US Patent 3,928,057 1975). These coatings are seen throughout the literature as either “porous” or “dense” coatings consisting of sodium silicate and or sodium aluminate. Almost every manufacturer has at least a couple of grades with durable microporous coatings.
