# Supplementary material for: Reliable nanomaterial classification of powders using the volume-specific surface area method
Source: J Nanopart Res. 2017 Feb 11;19(2):61. doi: 10.1007/s11051-017-3741-x (PMC5306339; doi:10.1007/s11051-017-3741-x)
Supplement: Supplementary file 1 — (DOCX 2598 kb) [file 11051_2017_3741_MOESM1_ESM.docx]

Reliable nanomaterial classification of powders using the volume-specific surface area method

Wendel Wohlleben,^[[1]](#footnote-1)#§^ Johannes Mielke,^[[2]](#footnote-2)#^ Alvise Bianchin,^[[3]](#footnote-3)^ Antoine Ghanem,^[[4]](#footnote-4)^ Harald Freiberger,a Hubert Rauscher,^[[5]](#footnote-5)^ Marion Gemeinertb and Vasile-Dan Hodoroabab^§^

Contents

[Summary 1](#_Toc469473755)

[Derivation of the quantity *dmin_VSSA_* 2](#_Toc469473756)

[Calculation of the uncertainty introduced by the aspect ratio cutoff values 3](#_Toc469473757)

[Calculation of multimodal material VSSA 4](#_Toc469473758)

[EM data for NanoDefine materials, including SEM method on platelets 6](#_Toc469473759)

[EM, BET, density data for the JRC/Eurocolour materials 9](#_Toc469473760)

[EM, BET, density data for the further real-world industrial materials 10](#_Toc469473761)

[Literature 12](#_Toc469473762)

# Summary

In the supporting information, the quantity *dmin_VSSA_* is derived and the influence of the particle aspect ratio cutoffs is evaluated. Furthermore, details are presented about the materials employed for the main article, including the sample preparation for the various measurements. Additional chapters describe the evaluation of the platelet thickness by SEM and the influence of multimodality on the classification by *VSSA*.

# Derivation of the quantity *dmin_VSSA_*

The JRC has introduced shape dependent *VSSA* cutoff values (Roebben et al. 2014), which allow classifying a material as NM, when its *VSSA* is above this cutoff and as non-NM when its *VSSA* is below. In this work, similarly to the approach by JRC, the minimal particle dimension *dmin_VSSA_* is calculated from the materials' *VSSA*. This quantity enables a direct comparison of the results to the EM-derived *d_50_* of the smallest particle dimension, which is more straightforward than using *VSSA* cutoff values and leads to the same classification results.

The property *dmin_VSSA_* is defined in the main article, equation 3 and Figure 1, as
${dmin}_{\text{VSSA}}(D) =\frac{2D}{VSSA}$ where *D* is the number of small dimensions. In this section it will be shown how it can be derived for several classes of particle shapes: spheres, cubes, rods and platelets.It is instructive to start with the case of a sphere (*D*=3) where $dmin$ is the spheres diameter. Its volume is $V=\frac{\pi}{6}{dmin}^{3}$ and its surface $S=\pi{dmin}^{2}$. Consequently, its *VSSA* is

$$VSSA=\frac{S}{V}=\frac{6}{dmin}$$

Therefore, a sphere with a diameter of 100 nm has a *VSSA* of 60 m^2^/cm^3^. When the *VSSA* is known from a measurement, it is possible to calculate $dmin$ by

$$dmin=\frac{6}{VSSA}$$

For general cuboids, the volume is $V=a b c$, the surface $S=2 (ab+bc+ac)$ and the *VSSA*:

$$VSSA=\frac{S}{V}=\frac{2}{a}+\frac{2}{b}+\frac{2}{c}$$

A cube (*D*=3) with equal sides ($a=b=c=dmin$) therefore has a *VSSA* of:

*D* = 3: $VSSA=\frac{S}{V}=\frac{6}{dmin}$

which is identical to the case of the sphere. For elongated particles (*D*=2), like fibers or tubes, the relation between the sides is $c\gg a=b=dmin$. In the limit of $c\to\infty$, the *VSSA* becomes

*D* = 2: $\lim_{c\to\infty} VSSA=\frac{2}{a}+\frac{2}{b}=\frac{4}{dmin}$

Analogously, the side relation for platelets is $dmin=b\ll c$ and $b\ll a$. In the limit of $a,c\to\infty$, the *VSSA* becomes

*D* = 1: $\lim_{a,c\to\infty} VSSA=\frac{2}{b}=\frac{2}{dmin}$

When generalizing the three cases for *D* = 1, *D* = 2 and *D* = 3, the following formula is obtained:

$$VSSA\left( D \right)=\frac{2D}{dmin}$$

Solving this expression for ${dmin}$ yields equation 3 in the main text:

$${dmin}_{VSSA}\left( D \right)=\frac{2D}{VSSA}$$

(when entering the $VSSA$ in units of m^2^/cm^3^, ${dmin}_{VSSA}$ is obtained in µm)

When no information about the particle shape is known, the particles are assumed to be spherical (*D*=3). In this case, it cannot be expected that the formula derived above still yields the minimum particle dimension, but instead a spherical equivalent diameter is calculated, which is designated $d_{VSSA}$ throughout this work:

$$d_{VSSA}=\frac{6}{VSSA}$$

(when entering the $VSSA$ in units of m^2^/cm^3^, $d_{VSSA}$ is obtained in µm)

It is important to keep in mind that the expressions derived here are only valid for single particles or perfectly monodisperse particle size distributions. Their applicability to materials with polydisperse particle size distributions as encountered in real-world industrial materials is the topic of the present study.

# Calculation of the uncertainty introduced by the aspect ratio cutoff values

The shapes considered in the previous chapter for *D* = 1, 2, 3, are convenient for calculating the ${dmin}_{VSSA}$ value, but can only be seen as an approximation for the real particle shape. Therefore, it is necessary to define the properties a particle needs to have in order to be attributed a certain *D* value and to assess its potential influence on the results.

In the main text it is proposed to select *D* for a given material according to the average aspect ratio (*AR*) of the particles.

*D* = 3 for particles with *AR* < 3:1
*D* = 2 for particles with *AR* > 3:1:1
*D* = 1 for particles with *AR* > 3:3:1

In the following, the largest possible influence of these cutoffs on the derived ${dmin}_{VSSA}$ is evaluated.

For *D* = 3, the maximum possible deviation from the equal sided cube occurs when the smallest side has a length of $dmin$ and the other two sides a length of $3dmin$. In this case the *VSSA* is:

$$VSSA=\frac{2}{dmin}+\frac{2}{3dmin}+\frac{2}{3dmin}=\frac{5}{9}\frac{6}{dmin}\approx0.56\frac{6}{dmin}$$

$$\overset{\Rightarrow}{} dmin=\frac{5}{9}\frac{6}{VSSA}\approx0.56\frac{6}{VSSA}$$

For *D* = 2, the maximum possible deviation as compared to the case of one dimension going to infinity is to have two sides with a length of $dmin$ and the third with a length of $3dmin$, hence:

$$VSSA=\frac{2}{dmin}+\frac{2}{dmin}+\frac{2}{3dmin}=\frac{7}{6}\frac{4}{dmin}\approx1.167\frac{4}{dmin}$$

$$\overset{\Rightarrow}{} dmin=\frac{7}{6}\frac{4}{VSSA}\approx1.167\frac{4}{VSSA}$$

For *D* = 1, the maximum deviation of the shape to the case of two dimensions going to infinity is to have one side with a length of $dmin$ and two sides with a length of $3dmin$:

$$VSSA=\frac{2}{dmin}+\frac{2}{3dmin}+\frac{2}{3dmin}=\frac{5}{3}\frac{2}{dmin}\approx1.67\frac{2}{dmin}$$

$$\overset{\Rightarrow}{} dmin=\frac{5}{3}\frac{2}{VSSA}\approx1.67\frac{2}{VSSA}$$

In Table S1, the here calculated maximum deviations induced by the *AR* cutoffs are compared to the value obtained by the simple approach when characterizing the particles only by *D* = 1, 2, 3. In all cases, the largest possible relative deviation is below 70%.

Table S1: Maximum deviation of dmin_VSSA_ induced by the aspect ratio cutoffs.

| *D* | *dmin_VSSA_* (*D*) | *dmin_VSSA_* (max deviation) | Relative deviation |
| --- | --- | --- | --- |
| 1 | $\frac{2}{VSSA}$ | $1.67\frac{2}{VSSA}$ | +67% |
| 2 | $\frac{4}{VSSA}$ | $1.167\frac{4}{VSSA}$ | +17% |
| 3 | $\frac{6}{VSSA}$ | $0.56\frac{6}{VSSA}$ | -44% |

Importantly, the here derived numbers are only the largest possible deviations due to the *AR* cutoffs. Other sources of uncertainty on the *dmin_VSSA_* are not considered in this evaluation.

# Calculation of multimodal material *VSSA*

The bimodal material also provides a good opportunity to test the predictions of the different *VSSA* definitions: the definition as in equation 1 (main text) and the number weighted *VSSA* (Lecloux 2015). One can use the number weighted particle size distributions of the individual materials as measured by EM and combine them into a bimodal PSD:

PSD for the individual materials:

- $q_{0f}(d)$: Number weighted PSD for fine BaSO_4_, normalized: $\int_{0}^{\infty} q_{0f}\left( d \right)dd=1$
- $q_{0uf}\left( d \right)$: Number weighted PSD for ultra-fine BaSO_4_, normalized: $\int_{0}^{\infty} q_{0uf}\left( d \right)dd=1$

Both of them are combined into the bimodal PSD $q_{0bi}\left( d \right)$ using $nr$, the number ratio, which has a value of 300 in the here presented case:

$$q_{0bi}\left( d \right)=\frac{{nr q}_{0uf}\left( d \right)+q_{0f}(d)}{nr+1}$$

The denominator ensures that $q_{0bi}\left( d \right)$ is again normalized. For the *VSSA* according to equation 1, the total surface and volume of the material need to be calculated separately (assuming spherical particle shape):

$$S=\pi\int_{0}^{\infty} d^{2}q_{0bi}\left( d \right)dd=\frac{\pi}{nr+1}\left( nr\int_{0}^{\infty} d^{2}q_{0uf}\left( d \right)dd+\int_{0}^{\infty} d^{2}q_{0f}\left( d \right)dd \right)$$

$$V=\frac{\pi}{6}\int_{0}^{\infty} d^{3}q_{0bi}\left( d \right)dd=\frac{\pi}{6}\frac{1}{nr+1}\left( nr\int_{0}^{\infty} d^{3}q_{0uf}\left( d \right)dd+\int_{0}^{\infty} d^{3}q_{0f}\left( d \right)dd \right)$$

Inserting the so obtained values in equation 1 yields in this case a *VSSA* of 24.6 m^2^/cm^3^.

The number weighted *VSSA* (Lecloux 2015) (assuming spherical particle shape) is the result of the following integral:

$$6\int_{0}^{\infty} \frac{1}{d}q_{0bi}\left( d \right)dd=\frac{6}{nr+1}\left( nr\int_{0}^{\infty} \frac{1}{d}q_{0uf}\left( d \right)dd+\int_{0}^{\infty} \frac{1}{d}q_{0f}\left( d \right)dd \right)$$

This integral predicts a number weighted *VSSA* of 309 m^2^/cm^3^.

# EM data for NanoDefine materials, including SEM method on platelets

Table S2: Sample preparation for EM measurements: SEM measurements were performed with a Zeiss Supra 40 between 5 kV and 20 kV using the *InLens* detector, only CaCO_3_ was imaged in the TSEM mode at 20 kV. For the platelet materials kaolin and nano steel, the first preparation leads to flat lying particles, the second to randomly oriented particles used for measuring the platelet thickness. TEM 1 is a FEI Tecnai G2 operated at 200 kV equipped with a field emission gun, operated in HAADF-STEM mode, only for the ultrafine BaSO_4_, BF-TEM was used. TEM 2 is a Hitachi HD2700 operated at 200 kV, STEM dark field imaging, only for the nano steel secondary electron imaging was used. The following stabilizers were used: Sodium hexametaphosphate (SHMP), Tetra-sodium pyrophosphate (TSPP) and Sodium Butyl Naphthalene Sulphonate (Nekal-BX).

| **Material** | **SEM** | **TEM 1** | **TEM 2** |
| --- | --- | --- | --- |
| organic pigment (transparent) | aqueous suspension, drop on carbon grid |  | aqueous suspension of Nekal-BX coated powder, alcian blue treated *formvar* grid on drop |
| organic pigment (opaque) | aqueous suspension, drop on carbon grid |  | aqueous suspension, alcian blue treated *formvar* grid on drop |
| BaSO_4_ (fine grade) | aqueous suspension, drop on carbon grid | aqueous, SHMP stab. suspension, drop on carbon grid |  |
| BaSO_4_ (ultrafine grade) | aqueous suspension, drop on carbon grid | aqueous suspension, spread drop between glas slides, dip hydrophilic carbon grid |  |
| MWCNT | aqueous suspension, drop on carbon grid | aqueous, BSA stab. suspension, dip carbon grid in suspension |  |
| Nano Steel | aqueous suspension, drop on carbon grid |  | aqueous suspension, alcian blue treated quantifoil grid on drop |
|  | Powder on conductive carbon pad or bulk Al, blow off |  |  |
| CaCO_3_ (fine grade) | aqueous suspension, drop on carbon grid | aqueous suspension, drop on carbon grid | aqueous SHMP stab. suspension, alcian blue treated quantifoil grid on drop |
| Kaolin | aqueous suspension, drop on carbon grid | aqueous, TSPP stab. suspension, dip carbon grid into suspension |  |
|  | Powder on bulk Al, blow off |  |  |
| coated TiO_2_ | Powder on bulk Cu, blow and wipe it off | aqueous, SHMP stab. suspension, drop on carbon grid | aqueous, SHMP stab. suspension, drop on carbon grid |
| Zeolite powder | Powder on carbon TEM grid, blow off visible powder |  | aqueous suspension, alcian blue treated quantifoil grid on drop |
| basic methacrylate copolymer | Powder on Si wafer, blow and wipe off visible powder |  |  |

Classifying platelet materials (*D* = 1) according to the EC recommendation (European Commission 2011) is a challenge even for EM, because the relevant smallest dimension is the particle thickness. When the particles are deposited on the substrate from a suspension, they tend to lie flat on the substrate and thus, their thickness cannot be determined from 2D EM images (Babick et al. 2016).

To demonstrate that the *dmin_VSSA_* for platelet materials (*D* = 1) yields a reasonable estimate for the platelet thickness, it was tried to measure the thickness of the two NanoDefine platelet materials (kaolin and nano steel) by high resolution SEM. To achieve this task, the powder was distributed on clean bulk Al or a conductive carbon pad and subsequently all loose powder was blown off. Thereby the particles retain a random orientation on the substrate and the SEM operator was asked to take images specifically of upright standing platelets, where the platelet thickness can be measured.


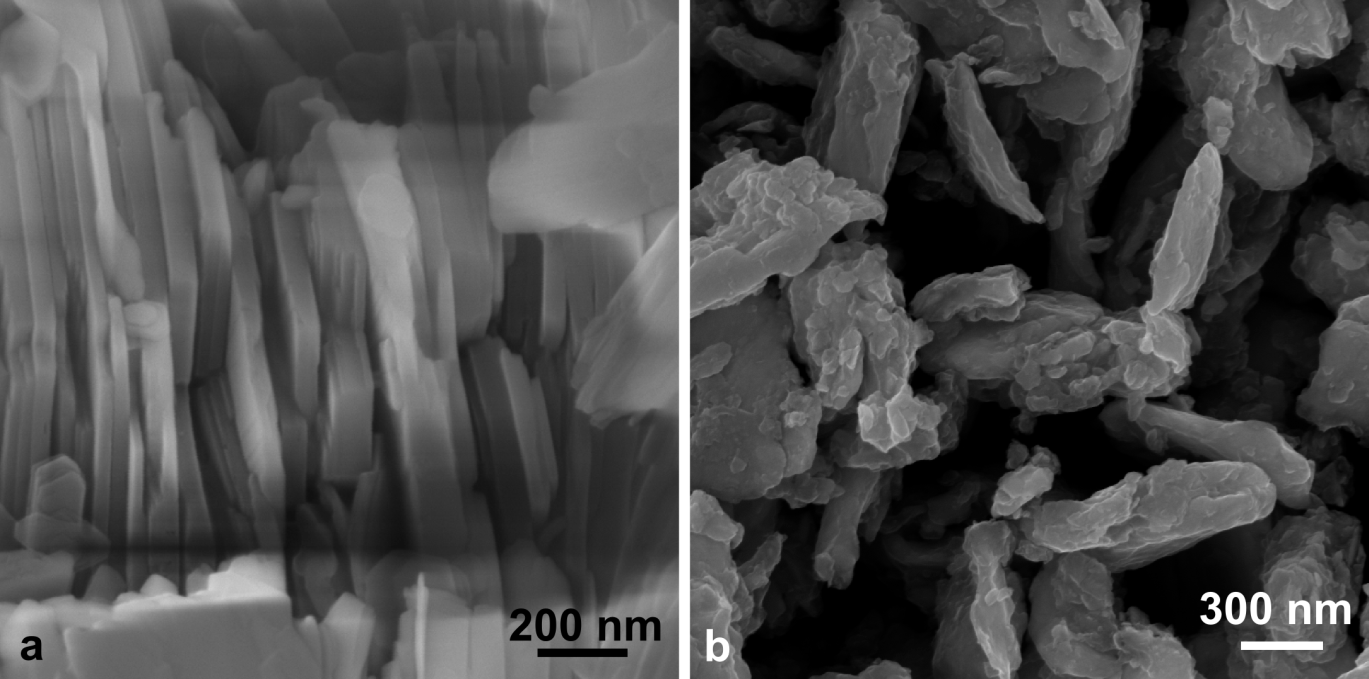


Figure S1: Evaluation of platelet thickness. SEM micrographs of upright standing kaolin platelets (a) and randomly oriented nano steel (b), both on a bulk Al substrate and recorded at 10 kV with an *InLens* detector. (c) Measured distributions of platelet widths for both materials, for nano steel on two different substrates.

In Figure S1 representative SEM images of both materials can be found together with the measured width distributions. Especially for the kaolin, the width could be determined accurately, due to its clearly defined structure, which makes it easy to identify single platelets and measure their width. For the nano steel, however, measuring the thickness is very difficult, due to its rather undefined shape and complicated surface structure, so that it is difficult to identify the appropriate dimensions in the micrographs. An overview of the so obtained results can be found in Table S3 and also in the overview Table 2 in the main article.

**Table S3:** Overview of the platelet thicknesses as determined in Fig. S1, containing the median width and standard deviation (*StD*) of every material together with the number of evaluated particles (*N*). In the case of nano steel, where measurements on two different preparations are available, both results are averaged.

| **Material** | **Median width**  **/nm** | ***StD***  **/nm** | ***N*** | **Mean**  **/nm** | ***StD***  **/nm** |
| --- | --- | --- | --- | --- | --- |
| **Kaolin on bulk Al** | 37.4 | 20.5 | 113 | 37.4 |  |
| **Nano steel on carbon tab** | 101.2 | 38.6 | 34 | 96.0 | 7.4 |
| **Nano steel on bulk Al** | 90.7 | 25.5 | 26 |  |  |

# EM, BET, density data for the JRC/Eurocolour materials

In a pilot round robin, BET and *VSSA* were reported on a series of fillers and pigments from eight laboratories throughout Europe (Pena et al. 2014). The results (Table S4) were reproducible within a relative standard deviation (*RelStDev*) of less than 20%, an exception being the material of lowest *SSA* (Cu/Zn pigment metal 2). TEM with manual, semi-automated or automated (only fumed SiO_2_) image evaluation of the smallest external diameter was performed as benchmark (i. e. only one analysis). Due to the complex shapes of the individual particulate materials, the authors noted a considerable ambiguity in TEM evaluation with respect to the selection of the smallest external dimension. One of the materials (Cu/Zn Pigment metal 2) is a platelet material and hence its smallest dimension could not be determined by EM. For two other materials (Fe_2_O_3_ Pigment Red 101 and CoAl_2_O_4_ Al-Co-Blue) the correct identification of the particles in EM micrographs was ambiguous due to the particles complex shape. Because of its insufficient EM evaluation quality, the Al-Co-Blue was excluded from the here presented comparison between *dmin_VSSA_* and the median *Feret_min_* by EM.

Table S4: *VSSA* (by BET) results on inorganic and organic fillers and pigments from the JRC/Eurocolour study (Pena et al. 2014) both below and above the cutoff.

| **Material** | ***SSA* (BET) (*n*=8)** | ***SSA* (BET) StDev** | **skeletal density** | ***VSSA* (BET)** | ***VSSA* (BET) *StDev*** | ***VSSA* (BET) *RelStDev*** | **Median *Feret_min_* (EM)** |
| --- | --- | --- | --- | --- | --- | --- | --- |
|  | **m²/g** | **m²/g** | **g/cm³** | **m²/cm³** | **m²/cm³** | **%** | **nm** |
| Fumed SiO_2_ | 209 | 7.7 | 2.2 | 459 | 16.9 | 4 | 12 |
| FeOOH Pigment Yellow 42 | 88.2 | 8.6 | 3.7 | 326 | 31 | 10 | 20 |
| TiO_2_ Rutile | 14.8 | 0.8 | 4.1 | 61 | 3.2 | 5 | 210 |
| Cu/Zn Pigment metal 2 | 4.7 | 1.4 | 7.7 | 36 | 10 | 28 | 4000 |
| Fe_2_O_3_ Pigment Red 101 | 8.8 | 0.5 | 5.0 | 44 | 2.2 | 5 | 249 |
| CoAl_2_O_4_ Al-Co-Blue | 7.8 | 0.3 | 4.2 | 33 | 1.4 | 4 | 527 |
| TiO_2_ Anatase | 9.1 | 0.4 | 3.8 | 35 | 1.4 | 4 | 130 |
| Azo Pigment Yellow 83 transparent | 58.7 | 11.4 | 1.5 | 86 | 17 | 20 | 47 |

# EM, BET, density data for the further real-world industrial materials

The test set for the proposed screening strategy consists of additional real-world industrial materials. EM images of those materials are shown in Fig. S2. The BET and density values were extracted from the manufacturers material data sheets. These data, together with the results of a manual EM analysis performed by the manufacturer, are presented in Table S5.


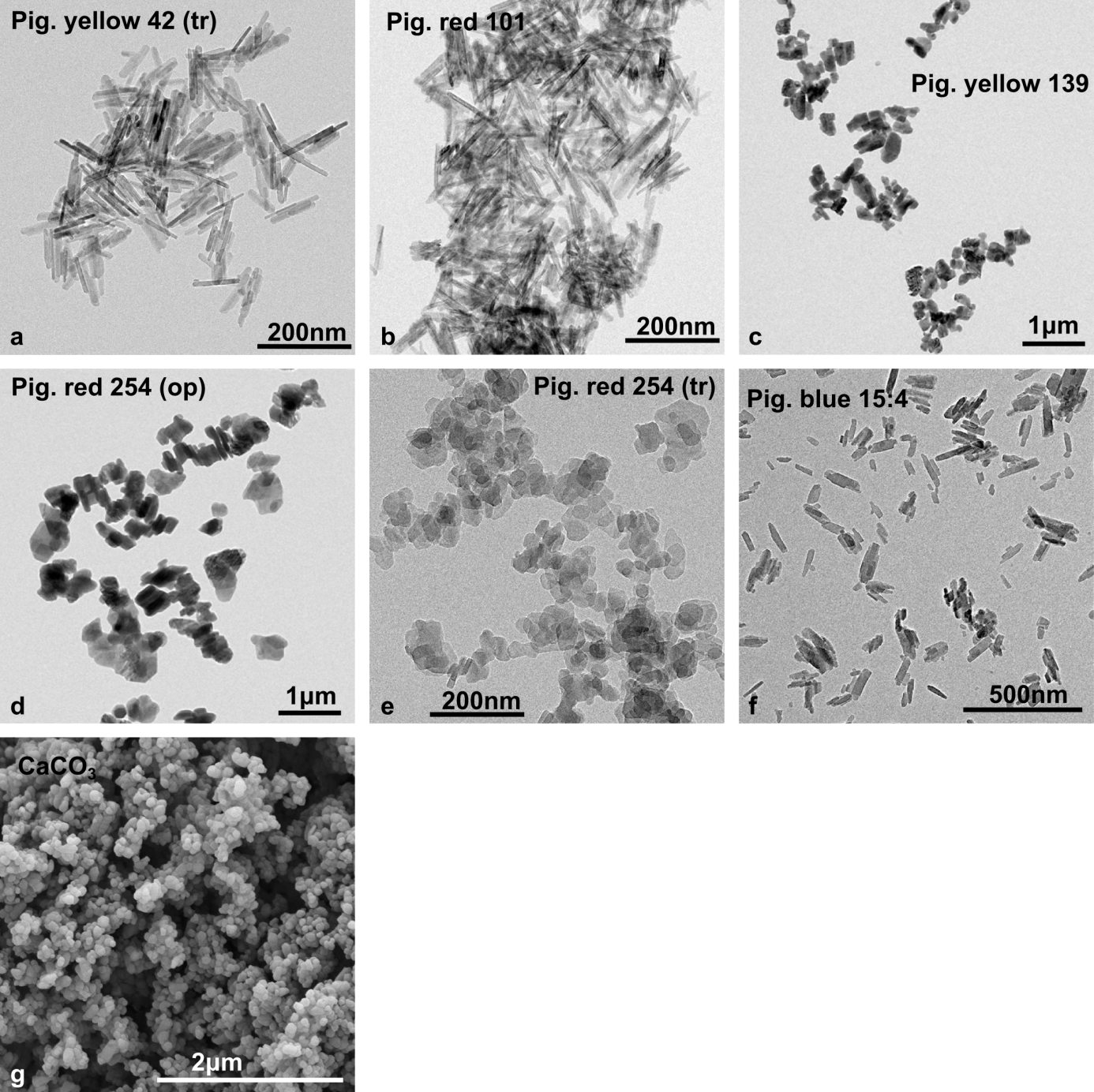


Figure S2: Electron microscopy images of the additional industrial test set materials. (a): Pigment yellow 42 (transparent), (b): Pigment red 101, (c): Pigment yellow 139, (d) Pigment red 254 (opaque), (e): Pigment red 254 (transparent), (f): Pigment blue 15:4, (g): CaCO_3_. Sample preparation and measurement conditions: (a) – (f): Material dispersed in ethanol, imaging with Field Emission Gun – Transmission Electron Microscopy (FEG-TEM), (g): Powder imaged as such with SEM.

Table S5: *VSSA* (by BET) results on further real-world materials, both below and above the cutoff, as well as the EM-derived median particle size.

| **Material** | ***SSA* (BET) (*n*=1)** | **skeletal density** | ***VSSA* (BET)** | **Median *Feret_min_* (EM)** |
| --- | --- | --- | --- | --- |
|  | **m²/g** | **g/cm³** | **m²/cm³** | **nm** |
| Pigment Yellow 42 (transparent) | 83 | 3.9 | 323.7 | 10.2 |
| Pigment Red 101 | 93 | 4.5 | 418.5 | 9.3 |
| Pigment Yellow 139 | 25 | 1.7 | 42.5 | 149.9 |
| Pigment Red 254 (opaque) | 15 | 1.63 | 24.5 | 233.3 |
| Pigment Red 254 (transparent) | 94 | 1.63 | 153.2 | 35.8 |
| Pigment Blue 15:4 | 64 | 1.61 | 103.0 | 30.0 |
| CaCO_3_ | 18 | 2.7 | 48.6 | 70 |

# Literature

Babick F, Mielke J, Wohlleben W, Weigel S, Hodoroaba V-D (2016) How reliably can a material be classified as a nanomaterial? Available particle-sizing techniques at work. Journal of Nanoparticle Research 18:158

COMMISSION RECOMMENDATION of 18 October 2011 on the definition of nanomaterial (2011/696/EU); 2011. Official Journal of the European Union.

Lecloux AJ. Discussion about the use of the volume-specific surface area (VSSA) as criteria to identify nanomaterials according to the EU definition. Journal of Nanoparticle Research. 2015;17(11).

Pena JBV, Kund K, Hempelmann U, Wohlleben W, Koch T, Burke A, et al. Basic comparison of particle size distribution measurements of pigments and fillers using commonly available industrial methods. Publications Office of the European Union; 2014.

Roebben G, Rauscher H, Amenta V, Aschberger K, Sanfeliu AB, Calzolai L, et al. Towards a review of the EC Recommendation for a definition of the term "nanomaterial" - Part 2: Assessment of collected information concerning the experience with the defintion. European Commission, Joint Research Centre; 2014.

1. BASF SE, Material Physics Research, 67056 Ludwigshafen, Germany [↑](#footnote-ref-1)
2. BAM – Federal Institute for Materials Research and Testing, 12205 Berlin, Germany [↑](#footnote-ref-2)
3. MBN nanomaterialia s.p.a., 31050 - Vascon di Carbonera – TV, Italy [↑](#footnote-ref-3)
4. Solvay - R&I Centre Brussels, 1120 Brussels, Belgium [↑](#footnote-ref-4)
5. European Commission, Joint Research Centre, Nanobiosciences Unit, 21027 Ispra, Italy

   ^#^ Equal contributions

   ^§^ Corresponding authors: wendel.wohlleben@basf.com, dan.hodoroaba@bam.de [↑](#footnote-ref-5)
